# Improving Access to Medicines for Neglected Tropical Diseases in Developing Countries: Lessons from Three Emerging Economies

**DOI:** 10.1371/journal.pntd.0001390

**Published:** 2012-02-28

**Authors:** Francesca Holt, Stephen J. Gillam, Jeremiah M. Ngondi

**Affiliations:** 1 St John's College, University of Cambridge, Cambridge, United Kingdom; 2 Department of Public Health and Primary Care, University of Cambridge, Cambridge, United Kingdom; René Rachou Research Center, Brazil

## Introduction

Neglected tropical diseases (NTDs) are those largely ignored by medical science, partly because they do not represent a viable commercial market for private pharmaceutical companies. These diseases are endemic in developing countries and have a significant impact at both personal and national levels. Globally, NTDs affect an estimated 2.7 billion people living on less than US$2 per day [1] and potently reinforce the poverty cycle [2]. At present, the prevailing strategy for improving access to medicines for these NTDs is drug donation programmes, which, despite providing some of the highest economic returns of public health programmes at 15%–30% [2], [3], have uncertain sustainability. Countries in demographic and economic transition are uniquely poised to be leaders in a shift towards a more sustainable, affordable means of providing access to medicines for NTDs.

## The Position of the Emerging Economies

China, India, and Brazil are three of the largest of more than 20 countries in economic and demographic transition commonly referred to as emerging economies [4]. Along with Russia, they are referred to as the “BRIC” countries and are predicted to overtake the G6 countries (United States, Japan, United Kingdom, Germany, France, and Italy) in terms of gross domestic product (GDP) by 2040 [5]. Their pharmaceutical industries are concomitantly booming; the pharmaceutical market in China is expected to drive US$40 billion in growth through 2013, whilst Brazil and India are each expected to add US$5–15 billion to the pharmaceutical market [6].

However, in terms of disease burden and health care, these countries continue to suffer many of the problems found in developing countries, including NTDs. Brazil is estimated to be afflicted by six of the NTDs, whilst China and India are thought to harbour four [7]. Furthermore, India, China, and the Americas account for 59% of the world population without access to essential medicines [8]. But these countries are unique in that they have managed to cultivate their own pharmaceutical industry with the capacity to produce affordable drugs for NTDs for use in domestic and international markets.

A review by Frew et al. identified 78 “innovative home-grown, small to medium-size health biotechnology companies in the emerging economies in Brazil, China, India and South Africa”. These “had a collective pipeline of nearly 500 products for more than 100 indications”. Of the products for HIV, malaria, tuberculosis, and NTDs, two-fifths (40.5%) of the products on the market and 62.9% of the products in development were for NTDs [9]. For example, Fungisome, a liposomal amphotericin B used to treat visceral leishmaniasis, was developed by an Indian company, Lifecare Innovations. Dermacerium, a topical agent used in the treatment or prophylaxis of skin infections in patients with leprosy, is produced by Silvestre Labs, Brazil [9]. Clearly, these countries recognise the worth of investment in developing medicinal tools to combat NTDs and have the capacity to do so.

## Experiences of India and Brazil

So how are the emerging economies working to improve access to medicines for NTDs? Lower costs are helping to stimulate the development of medicines in these emerging economies. The cost of research and development (R&D) and manufacturing in India and China are estimated to be one-eighth and one-fifth of the costs incurred by Western companies, respectively. This is attributed to lower fixed asset costs (i.e., lower costs of building manufacturing facilities), cheaper labour, lower costs of regulation, efficient manufacturing processes, a large suitable population to be recruited quickly and cheaply for clinical trials, and inexpensive marketing [10].

In India, the pharmaceutical industry grew through utilisation of low R&D costs and its building of generic drug production facilities. Later, when forced by the Agreement on the Trade-Related Aspects of Intellectual Property Rights (TRIPS), it moved towards innovative drug production. Brazil went through a similar process of utilising low production costs and expanding its generic drug industry. However, there was a key difference: the manner in which it dealt with the introduction of the TRIPS by the World Trade Organization (WTO). Here, we see how government technological and industrial policies have proved extremely influential in stimulating R&D [11].

Under the TRIPS Agreement, members of the WTO have to provide patent protection for inventions, which includes medicines and their methods of manufacture. Patent protection has to last at least 20 years from the date the patent application was filed [12]. The TRIPS Agreement permitted a transition period of 10 years in which developing countries could adapt. During the transition, a “mailbox” for patent deposits was set up. This system allowed patent deposits before the agreement to be placed in the “mailbox” and held there for the transition period before a decision need be made. India made full use of this transition period. The Indian Patents Act of 1970, which granted patent rights only to manufacturing processes, prevailed throughout the transition, until 2005. This act allowed Indian pharmaceutical companies to perform reverse engineering of branded drugs and sell them as generic drugs. The mailbox system also meant that Indian companies were able to predict which drugs were waiting for patent and adapt accordingly. When it came to the end of the transition period, patents were only granted in cases where there was no local production. Alongside policies to support development of local pharmaceutical companies such as high import tariffs, restriction of foreign direct investment, and price control, the way in which they adapted to the TRIPS Agreement allowed greater development of the national pharmaceutical industry [11].

Brazil, however, became fully compliant with the agreement after 2 years (by 1996). Once the agreement was implemented, Brazil began granting patents. Every depositor who had obtained a patent in another country could request one in Brazil without assessment. This pipeline mechanism also allowed a retroactive examination of patent deposits from products that only became patentable after the enactment of the new law [11].

The results of these different government policy decisions are marked. During the transition period, Brazil saw a decrease in its national market, whereas India had fostered a significant increase. Whilst Brazil was in negative trade balance for pharmaceutical and medicinal products, India was in positive trade balance and had achieved near self-sufficiency in most drugs and pharmaceuticals [13]. The multinational corporations with their “persistent hegemony” in the Brazilian market benefited from the application of TRIPS to Brazil, but the Brazilian companies did not [14].

As described by Morel et al., Brazil is trying to make up for lost ground with their new legal and regulatory framework, which includes the Law of Technological Innovation [15], and their Biotechnology Development Policy. These policy and legal changes are helping to generate new drugs to treat NTDs by bridging the gap between basic research and drug development. For example, the Brazil Department for Science and Technology and Ministry of Science and Technology together invested US$10 million in 76 peer-reviewed projects as part of a pilot R&D programme in 2007–2008 that aims to tackle dengue fever, Chagas' disease, leprosy, malaria, tuberculosis, and leishmaniasis. The programme builds on existing international networks of research and aims to strengthen capacity for research on NTDs, particularly in regions of Brazil where these diseases are endemic [13]. Furthermore, much can be learned about improving access to medicines from the successes of the Brazilian efforts to guarantee free antiretrovirals for patients with HIV/AIDS. Whilst local production of antiretroviral drugs was paramount, where this was not possible the drugs were imported as cheaply as could be negotiated. This was made possible by judicious use of compulsory licensing as a bargaining tool, and later by actual issue of a compulsory license [16], [17].

## Lessons and Their Application

From observations of the experiences of emerging economies such as India and Brazil, it can be seen that developing a competitive national pharmaceutical industry has the potential to ensure sustainable and affordable development of drugs for NTDs. These observations also underscore the importance of managing intellectual property and laws that support innovation.

Capacity for achieving comparable improvement in access to medicines in other emerging economies is as yet an untapped resource. It has been suggested, by Frew et al., that a “Global Health Accelerator” is needed to scale up the product development efforts of these emerging-economy firms [9]. The accelerator would create a shift away from the less sustainable business of product development at high costs followed by charitable donation, and towards affordable innovation. The accelerator model would utilise push and pull mechanisms to incentivise drug development, as well as support small firms in international business issues such as understanding regulatory environments, assessing markets, positioning products, identifying distribution channels, accessing financing, and identifying international commercialisation partners. It would also facilitate collaborative endeavours including public–private partnerships [9]. However, such ideas must go beyond the emerging economies. It is crucial that the lessons about stimulating innovation are shared effectively with the developing world.

There are early signs of efforts to scale up nascent drug R&D in developing nations. The first meeting of the new African Network for Drugs and Diagnostics Innovation (ANDI) was held in October 2008. The goal of the ANDI is to develop locally sustainable health R&D coordinated through an African-based and -led organisation [18], [19]. Similarly, the Initiative to Strengthen Health Research Capacity in Africa (ISHRECA) “seeks to promote the creation of self-sustaining pools of excellence capable of initiating and carrying out high quality health research in Africa as well as translating research products into policy and practice through better integrated approaches of capacity building at individual, institutional and system levels” [20].

Superimposed on these Africa-based organisations, The South-South Initiative works to promote collaboration between disease-endemic countries across Africa, Latin America, and Asia in the application of scientific, technological, and methodological advances to infectious diseases of poverty [21]. As exemplified by the investment made by Cipla, a large Indian pharmaceutical company, in the manufacture of antiretrovirals by Uganda's Quality Chemicals Industries, such collaborations are already helping tackle access to medicines for HIV. In this case, the viability of such collaboration was provided by advanced market commitments made by the Ugandan government as well as the promise of a market across the whole of Eastern Africa [22]. The beginnings of similar collaborative initiatives for NTDs can be seen. For example, a research network linking Brazil with Ghana, Nigeria, Angola, and Mozambique has been set up so that research and experience on NTDs can be shared [23].

## Conclusion

Currently, the dominant strategy for ensuring access to medicines for NTDs is drug donation from Western pharmaceutical companies. But this dependence upon profit-driven organisations is precarious. Clearly, a more sustainable approach is required. Unlike many developing countries, the emerging economies not only have a large NTD burden, but they are also beginning to show us a means of improving access to medicines for NTDs in a more sustainable fashion. They are developing their own policies of innovation, their own pharmaceutical industries, and their own medical solutions to NTDs. Their experiential knowledge is surely invaluable and can help to guide developing countries towards sustainable strategies to control NTDs.
